# Validation of a risk perception questionnaire developed for patients with rheumatoid arthritis

**DOI:** 10.1371/journal.pone.0219921

**Published:** 2019-07-22

**Authors:** Irazú Contreras-Yáñez, Pilar Lavielle, Patricia Clark, Virginia Pascual-Ramos

**Affiliations:** 1 Immunology and Rheumatology Department, Instituto Nacional de Ciencias Médicas y Nutrición Salvador Zubirán, Mexico City, Mexico; 2 Research Unit in Clinical Epidemiology, Hospital de Especialidades, Centro Médico Nacional Siglo XXI, Instituto Mexicano del Seguro Social, Mexico City, Mexico; 3 Clinical Epidemiology Unit, Hospital Infantil de México Federico Gómez, Mexico City, Mexico; 4 Medicine Faculty, Universidad Nacional Autónoma de Mexico, Mexico City, Mexico; Chinese Academy of Medical Sciences and Peking Union Medical College, CHINA

## Abstract

**Background:**

Risk perception is a multidimensional phenomenon that describes the individual’s judgment of the likelihood of experiencing something unpleasant. Risk perception helps to understand how rheumatoid arthritis patients perceive disease-related-risks. We developed and validated a risk perception questionnaire for Spanish speaking rheumatoid arthritis patients.

**Methods:**

The questionnaire development and validation was performed in 3 steps, using respective convenience samples. Step-1 included the conceptual model construction, 20 patient’s interviews to identify components from the conceptual model-dimensions and 11 healthcare provider´s consultations who identified RA related manifestations/complications (network and frequencies analysis). Step-2 consisted of item generation and reduction and questionnaire feasibility (n = 100). Step-3 consisted of the questionnaire psychometric validation (n = 270), which included content, face, construct (exploratory factor analysis) and criterion validity (logistic regression analysis) and consistency and stability (Cronbach’s α and test-retest).

**Results:**

Samples were representative of typical RA outpatients. Initial conceptual model included 7 dimensions, 3 for probability and 1 each, for responsibility, prevention, control and for severity (Step-1). The final version was considered feasible by the patients and included 27 items (Step-2). A five-factor model was most appropriated and resulted in 68.8% of the variance explained: Cronbach’s α = 0.90, intraclass-correlation-coefficient = 0.93 (95% CI = 0.90–0.95). A positive relation between number of external criteria from the charts and risk perception was found; all items had ≥80% agreement from experts; patients agreed about item´s semantic clarity (89%) and format adequacy (97%), (Step-3).

**Conclusions:**

The risk perception questionnaire was valid and reliable to evaluate risk perception construct in RA outpatients; it can be incorporated to routine care and clinical research, and guide interventions to improve patient’s health behaviors.

## Introduction

Rheumatoid arthritis (RA) is the most prevalent chronic inflammatory arthritis. The disease exhibits a worldwide distribution and primarily affects middle-aged women [1, 2]. Joint swelling and pain, substantial fatigue and a wide variety of extra-articular manifestations characterize the disease, which results in a progressive restriction of the patient’s functional capacity and a reduced quality of life [3, 4]. Increasing evidence emphasizes the impact of comorbidities during the natural course of the disease [5]. RA patients exhibit an additional increased risk of hospitalizations due to severe infections, cardiovascular and pulmonary complications and orthopedic surgery [6]. In addition, patients present increased mortality compared to paired controls, and cardiovascular complications account for most of this increased risk [7]. The progressive nature of the disease and its onset early in mid-life results in most patients living with the disease for 30 years or more, with considerable personal, social and economic impacts [8]. RA is a potentially disabling disease although earlier and aggressive treatment with disease modifying drugs (DMARDs) increases the likelihood to impact positively patient´s outcomes.

The updated 2016 European League against Rheumatism (EULAR) treatment-recommendations highlights that patient´s treatment must be based on shared decisions between the patient and the rheumatologist [9]. The principle underlying this recommendation is to have an effective patient-physician communication, which can be better achieved with the incorporation of the patient’s interests, perceptions, judgments and beliefs into the clinical discourse regarding therapeutic plans. Therapeutic proposals may be more effectively achieved and maintained if these proposals are perceived as patient-centered and patient-adopted.

Risk assessment is an intellectual discipline designed to aid in the identification, characterization and quantification of risks [10]. Humans have the ability to sense and avoid harmful environments, to codify and learn from experiences, and also to alter their environment and to respond to it; these capacities both create and reduce risks [11]. The adoption of health behaviors is associated with the recognition of risks [12]. Risk perception (RP) is defined as a multidimensional phenomenon that describes the individual’s judgment of the likelihood of experiencing something unpleasant [13]. Researchers adopted and applied the concept of RP to health [14] and suggested that the average RP level for a threat is related to the average level of the perceived characteristics of that particular threat, such as prevalence, controllability, preventability and seriousness [14–16]. RP is also associated with unfavorable health behaviors due to judgments errors, such as unrealistic optimism and unrealistic pessimism, which are defined as judgment biases in which subjects underestimate or overestimate the likelihood of experiencing a negative event related to health [17–19].

Most of the RP published literature focused on health behaviors that are driven to prevent the development of infectious diseases, such as AIDS [20], and specific cancers [21, 22] and facilitate the diagnosis of diabetes mellitus (DM) in healthy (at risk) individuals [23–25]. Two studies assessed RP in adult patients who were already diagnosed with type 2 DM, and both studies found an association between the degree of risk and adherence to medication [24, 25]. There is limited RP-literature published in the field of rheumatic diseases. Two qualitative related studies developed in French RA patients assessed most frequent RA-related fears [26, 27]; nonetheless, both terms, fear and risk are not equivalent, as “fear” is a feeling induced by a perceived danger or threat, an emotion; meanwhile, “risk” is a cognitive process, the threat of a quantifiable damage [28]. Ultimately, fear has been accepted as an aspect associated with RP [29, 30].

RP describes patient’s judgments, but there is no current validated instrument to assess RP in RA. The recognition of a significant risk to health in the face of the threat of complications can motivate patients to adopt preventive health behaviors. Rheumatologists should provide patients with information that brings the medical vision closer to the patient’s perception of disease-related-risks; in the clinical context of RA patients, physicians prioritize on adherence to disease-modifying anti-rheumatic drugs, as it has been related to better outcomes; nevertheless, non-compliance with treatment is a universal phenomenon which has been associated to the use of alternative medicine; in addition, RA patients are frequently recommended smoke cessation, healthy diets and the management of relevant comorbid conditions such as hypertension, serum lipid normalization and periodontitis. All of these require an active behavior involvement of the patients, which may be more easily achieved if patients perceived some life-styles as risk factors for unfavorable outcomes. Finally, assessing perceived risk may help explain how RA patients integrate their ideas concerning the disease and its treatments, and how this understanding affects their self-care management. Having these important issues in mind, the objective of the present study was to develop and validate the RP Questionnaire (RPQ) for Spanish speaking patients with RA.

## Material and methods

The study was performed in three steps: 1) Construction of a conceptual model of the RPQ; 2) Item generation and reduction to ensure feasibility, relevance and comprehension of items and instructions, scaling responses and scoring; and 3) Psychometric validation.

We followed the questionnaire construction process suggested by Streiner for health measurement scales when a current measure does not exist: Item generation; item testing and retesting, questionnaire reliability and validity [31].

### Description of samples

Three different convenience samples of consecutive RA patients were included. All patients were recruited from the RA outpatient clinic of the Instituto Nacional de Ciencias Médicas y Nutrición Salvador-Zubirán (INCMyN-SZ), a tertiary care level and national referral center for rheumatic diseases. All the patients had the diagnosis of RA, according to their primary rheumatologist criteria.

The first sample included 20 patients with longstanding disease, who had completed their participation in a clinical trial with an approved drug for RA and had disease activity score on 28 joints (DAS-28) available; those patients with low disease activity or remission (DAS28<3.2), who were at risk of a flare due to study drug discontinuation, were selected for the construction of a conceptual model of RP in RA. The second sample included 100 patients (in whom different drafts of the RPQ were applied), and was used for item generation and reduction. The last sample included 270 patients, and was used for the psychometric validation.

### Steps and procedures

#### Step 1: Construction of the conceptual model of the RPQ / literature review

Two authors reviewed the literature and identified: 1) Tools to assess RP in RA patients, and/or in patients with additional diagnosis; 2) Potential evaluative dimensions of the RP construct, and 3) Every manifestation/complication of RA and their corresponding frequency in different populations of RA patients.

#### Step 1: Construction of the conceptual model of the RPQ / patient and healthcare provider’s interviews

Semi-structured interviews were used in order to identify components to be included in the dimensions considered in the conceptual model [32]. A female social worker, trained and experienced in RA, performed personal and semi-structured, one-to one interviews, in a private room, for approximately 20 minutes each, to 20 RA patients which characteristics had been previously described. The interviewer asked open-ended questions about the likelihood of harms related to RA, and the clinical context of eventual flares because of stopping a disease specific medication, was given; components were derived from theses interviews, “Guide for semi-structured interviews” S1 Appendix. In addition, 6 rheumatologists and 5 RA-experienced physiotherapists were asked to list and rate (according to severity and frequency) 15 RA-related components (symptoms, manifestations and/or complications). Emphasis was made to consider long-term disease follow-up.

#### Step 2: Item generation and reduction / development of items and item´s reduction

Three types of sources were considered for item´s generation: Theory, key informant interviews and expert opinions (as described). In order to achieve sufficient redundancy prior to the reduction, the first draft (v.1) of the RPQ included 4 versions per each component selected according to the conceptual model (conceptually identical and structurally different) that resulted in 108 items; all were suggested by the social worker, who considered the wording used by the patients during the interviews. Then, 2 additional researchers (1 psychologist and 1 rheumatologist) reviewed the items, proposed corrections and reached a consensus of the updated draft, which was applied to 50 outpatients. Thereafter, a second updated draft (v.2) was obtained, limited to 54 items (2 versions per each of the 27 original items) and was applied to 50 additional RA outpatients; a final draft (v.3) of the RPQ, reduced to 27 items was obtained, “Final draft of the RPQ” S2 Appendix. The 27 items were ultimately distributed into 5 dimensions following the conceptual construction. During the reduction process, the homogeneity of the items was tested until the best inter-item and item-total correlation were achieved.

#### Step 2: Item generation and reduction / scaling responses

The basic level of formal education from our patients and their potential hand disability were considered. We selected a direct estimation method of responses, on a visual analogue scale (VAS). The VAS consisted of a 100-millimeter straight line with a verbal description at the endpoints. “No likelihood” appeared at the bottom of the scale, and “Absolute likelihood” appeared at the top of the scale. Patients were directed to mark on the line a point between the 2 endpoints, “Final draft of the RPQ” S2 Appendix.

#### Step 2: Item generation and reduction / item scoring

We used the method of standard scores to be able to compare our results with those eventually described in other populations. The 27 individual items were scored as the length of the VAS measured from the beginning of the line (left side) to the point marked by the patient (0 to 100 mm) for all the 100 patients included in sample 2. The mean of the 27 items was calculated, and a linear z transformation was applied to obtain a t-score [31].

#### Step 2: Item generation and reduction / feasibility

Feasibility was tested according to the following criteria: Time required to fill the scale, patients perceived item´s clarity and patient´s format acceptance.

#### Step 3: Psychometric validation / internal consistency and reliability

Internal consistency and reliability were determined. In order to assess temporal stability, RPQ was applied to 50 patients, twice, within a 1±1 week interval.

#### Step 3: Psychometric validation / validity

Judgment experts determined face and content validity. The validation group was integrated by 2 rheumatologists and 1 psychiatrist who reviewed relevant RP-literature. Each expert, blinded to other´s evaluation, rated each one of the 27 items included in the final version of the RPQ, according to the presence or absence of relevance, adequate wording, appropriate language and meaning. Construct validity was evaluated for each questionnaire dimension using factor analysis. Criterion validity was based on external criteria that were a priori defined for each of the 5 dimensions conforming the RPQ final model and that were recorded on patient´s charts.

### Statistical analysis

For step 1, the responses provided by patients and healthcare providers were analyzed using the modified natural semantic network technique [33], and elements above the breakpoint of Catell (asymptote of the accumulated frequency curve) were introduced in the preliminary version of the instrument [34].

For step 2, the homogeneity of the items was tested with the inter items correlation and the items’ contribution to the total score. Items with correlations <0.3 were discarded, based on the consideration that they were measuring something different from the scale [31].

For step 3, descriptive statistics was performed to estimate the frequencies and percentages (categorical variables) or the means and SD (continuous variables) of the sociodemographic and clinical characteristics of the main sample. Face and content validity by experts was examined with agreement percentage. Cronbach’s α and inter-item correlation for the complete scale and for each dimension was used to assess the internal consistency of the questionnaire. Cronbach's α interpretation was as follows: <0.70 indicates that individual items provide an inadequate contribution to the overall scale and values of >0.90 suggest redundancy [35]. For test–retest, intra-class correlation coefficients (ICC) and their 95% confident intervals (CI) were calculated based on a single measurement, absolute-agreement, 2-way mixed-effects model. According to the ICC, values <0.5 indicate poor reliability, between 0.5–0.75 moderate reliability, between 0.75–0.9 good reliability and values >0.9 indicate excellent reliability. Finally, 95% CI estimates between 0.83–0.94 were considered as good reliability level and those between 0.95–0.99 estimates, as excellent reliability level [36]. Floor and ceiling effects were determined as the percentage of patients who achieved the lowest and highest score of the scale, respectively. Construct validity was evaluated using exploratory factor analysis (principal components) with Varimax rotation. Sampling adequacy was confirmed using the Kaiser-Mayer-Olkin (KMO) (appropriate value ≥0.5) measure, and the use of factor analysis was supported by Bartlett's test of sphericity (significant value p<0.05). The number of factors was determined as the number of eigenvalues >1. The item-factor membership was determined by the factor loading as an indication of the degree to which each item was associated with each factor [37].

For criterion validity, the presence of each external criteria was compared between patients with and without RP, defined when the score of the RPQ was ≥ 61.7 mm (which corresponded to the 75^th^ percentile). Finally, the number of external criteria recorded from the charts was obtained for each patient and its association with RP was examined through logistic regression models.

Sample size was based on the methodological recommendations, which suggested a minimum of 50 patients for assessing construct validity, a minimum of 100 patients for assessing internal consistency, and 5 to 10 patients for each item of the instrument [38, 39].

All statistical analyses were performed using Statistical Package for the Social Sciences version 21.0 (SPSS Chicago IL). A value of p<0.05 was considered statistically significant.

### Ethical considerations

The study was performed in compliance with the Helsinki Declaration. Internal Review Board, “Comité de Ética en Investigación del Instituto Nacional de Ciencias Médicas y Nutrición Salvador-Zubirán” approved the study (Reference 1909). All participants provided written informed consent.

## Results

### Population characteristics (Table 1)

**Table 1:** Description of the sample´s characteristics.

	N = 20 (Step 1) (Conceptual model construction)	N = 100 (Step 2) (Item generation and reduction)	N = 270 (Step 3) (Psychometric Validation)
N° (%) of females	19 (95)	95 (95)	262 (97)
Years of age	52 (45–50)	53.5 (42–63)	57 (50–63)
Years of formal education	9 (9–12)	9 (9–11)	7 (7–9)
N° (%) of patients with medium-low SE level	19 (95)	90 (90)	251 (93)
Years of disease duration	20 (16–24)	13 (7.9–21.5)	12 (7–18)
N° (%) of patients with early disease (≤5 years)	0	19 (19)	45 (16.7)
N° (%) of patients in remission status	12 (60)	Not available	146 (54)
CRP, mg/dL	0.42 (0.15–1.1)	Not available	0.59 (0.21–1.9)
ESR, mm/H	8 (3–16)	Not available	14 (7–28)
N° (%) of patients with RF	20 (100)	Not available	240 (89)
N° (%) of patients with ACCP	20 (100)	Not available	170 (74)*
N° (%) of patients with major comorbidities	2 (10)	23 (23)	57 (21)
N° (%) of patients with joint replacement	4 (20)	12 (12)	43 (16)

Data as presented as median (25^th^-75^th^ interquartile range) unless otherwise indicated. N° = number; SE = socioeconomic; CRP = C reactive protein; ESR = erythrocyte sedimentation rate; RF = rheumatoid factor; ACCP = antibodies to cyclic citrullinated peptides.

*Data limited to 230 patients with sample available.

The 390 patients included in the study were divided in 3 samples and had similar characteristics. Patients were primarily women, as described in the Latin American region [40, 41], in their fifth decade of life, with basic formal education and medium low socioeconomic status (with at least 70% subsidy on the actual cost of their medical care). The population studied had long-standing disease, but patients with early disease (≤5 years of disease duration) were also represented.

In the sample for the psychometric validation (step 3), more than half of the patients were in remission and had acute reactants phase determinations (erythrocyte sedimentation rate [ESR] and C reactive-protein [CRP]) within normal range; also, the majority of the patients had rheumatoid factor (RF) and antibodies to cyclic citrullinated peptides (ACCP). These results were reproduced in the sample used during Step 1.

Finally, in the 3 samples, patients with a major comorbid condition and patients with a surgical joint replacement were also represented.

Patients and disease characteristics were similar in the 3 samples, but patients from the conceptual model construction sample (N = 20) had more often longer disease duration when compared to their counterparts (p≤0.03 for both comparisons).

### Construction of the conceptual model of the RPQ (Step 1)

Literature review did not identify RP questionnaire/scale in RA patients. Published conceptual models describe 5 dimensions (probability, responsibility, prevention, severity and control) [11–12, 14, 19].

**Fig 1:**
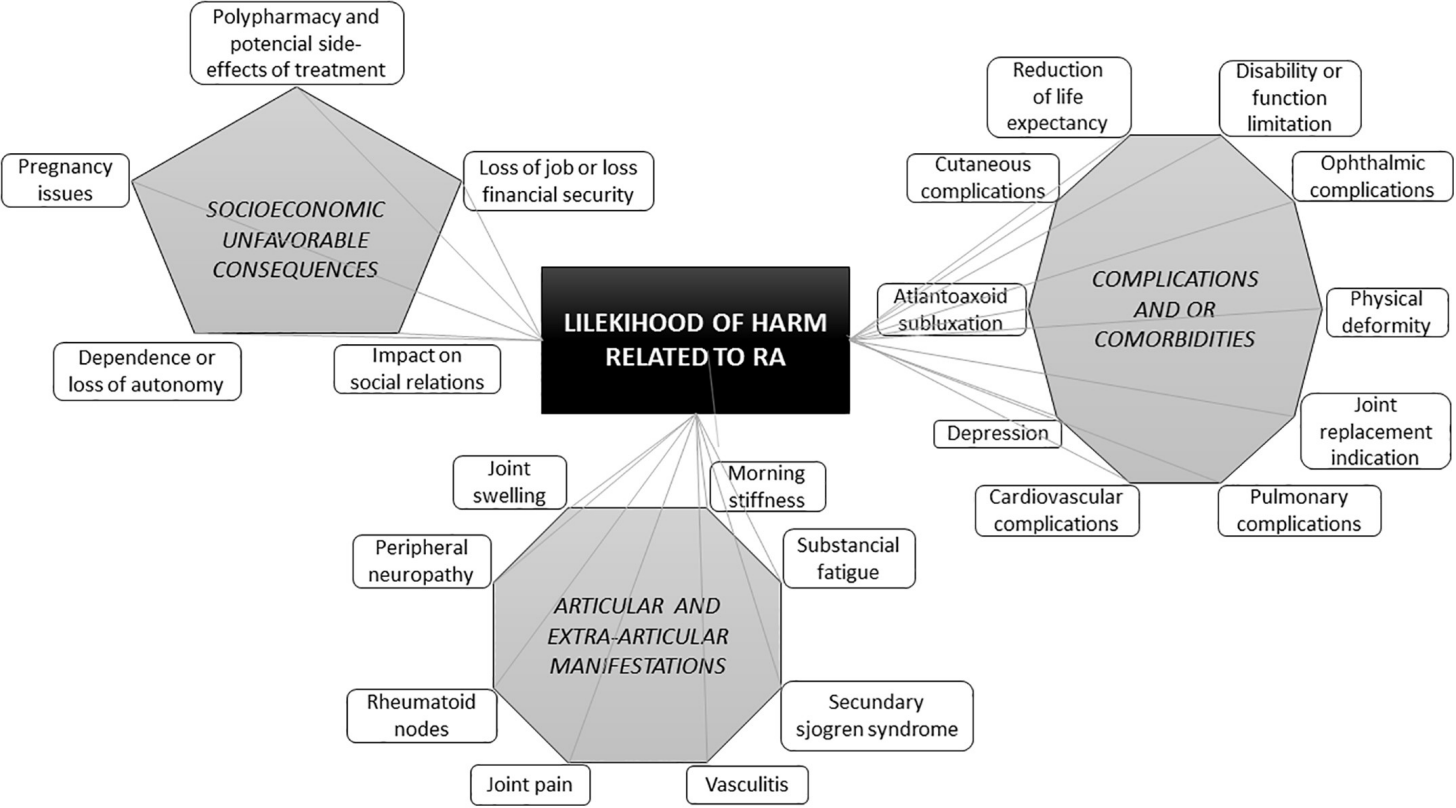
Semantic network graph for rheumatoid arthritis related articular and extra-articular manifestations, complications and/or comorbidities and socioeconomic unfavorable consequences. The figure is a schematic representation of the resulting network from the semantic network analysis. At the middle, the central concept and to the sides the network of objects extracted from patients interviews and healthcare providers and associated as related concepts.

Semantic network technique identified 13 components from patient interviews and 66 components from healthcare providers and all pertain to the probability dimension; based on the frequency of these, 23 components were selected. The 23 different RA-related manifestations and complications (8 from the patients and 15 from healthcare providers) were included in the probability dimension and separated in 3 sets of components, named as follows: Articular and extra-articular manifestations, complications and/or comorbidities and socioeconomic unfavorable consequences (Fig 1).

We used this conceptual framework and adapted to the RA context; the resulting model included same evaluative dimensions; nonetheless, the probability dimension was integrated by 3 sub-dimensions (patient likelihood to develop RA-related articular and extra-articular manifestations, patient likelihood to develop RA-related complications and/or comorbidities and patient likelihood to develop RA-related socioeconomic unfavorable consequences); the four additional dimensions were responsibility (patient perception of personal responsibility to develop complications), prevention (patient perception of the possibility to prevent RA-related complications), severity (patient perception of disease severity) and control (patient perception of personal control over the disease).

**Fig 2:**
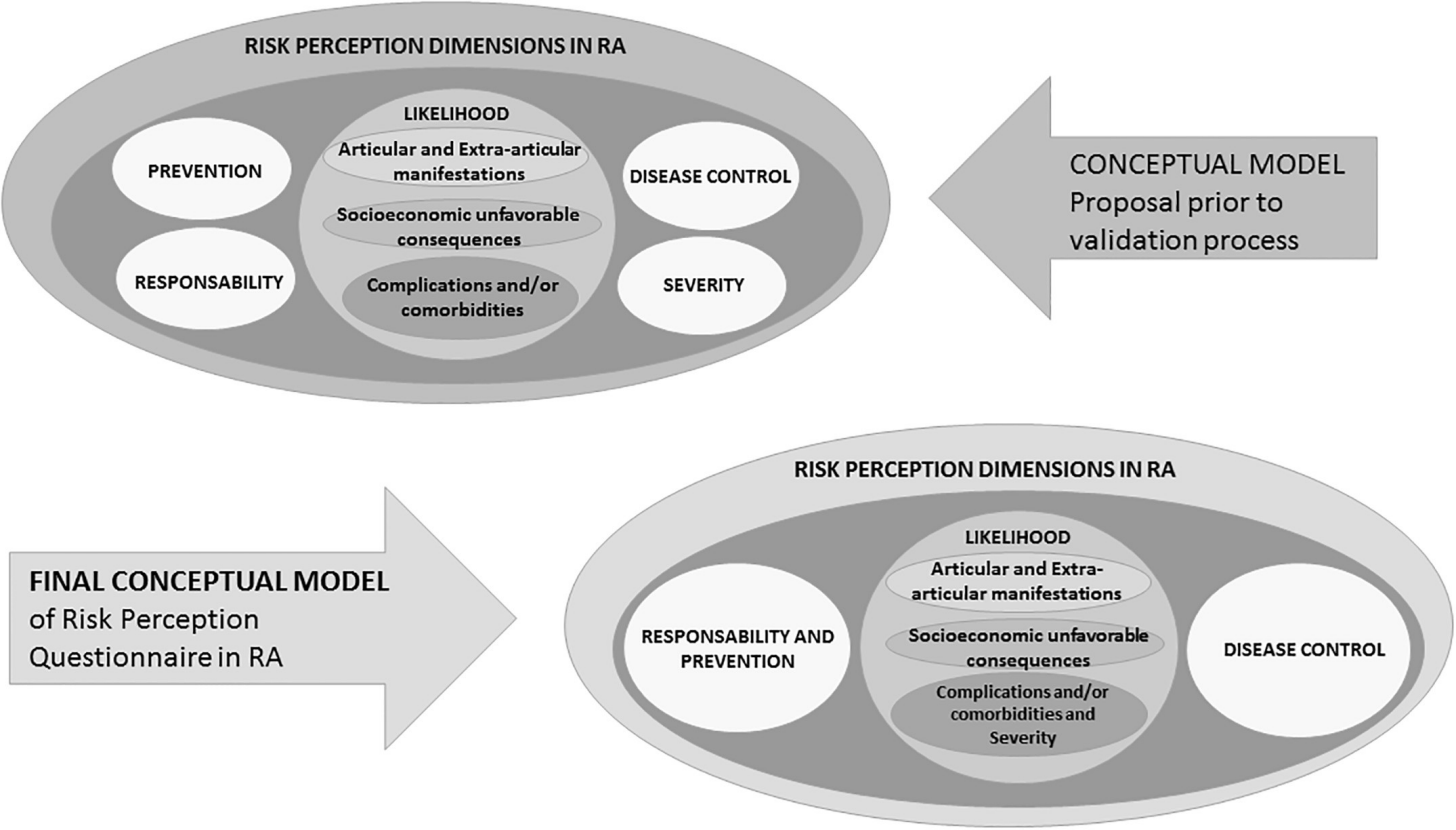
Theoretical models of risk perception dimensions. The figure shows in the left oval, the initial conceptual model and in the right oval the final conceptual model constructed with the results of the factor analysis.

After factor analysis (see below), this tentative conceptual model was not confirmed and the model finally proposed included the following 5 dimensions: Likelihood to develop RA-related articular and extra-articular manifestations, likelihood to develop RA-related complications and/or comorbidities and disease severity, likelihood to develop RA-related socioeconomic unfavorable consequences, patient perception of personal responsibility to prevent and develop complications and patient perception of personal control over the disease (Fig 2).

### Item generation and reduction (Step 2)

#### Item generation

All items produced less than 5% missing responses, and all were included in the analyses. Each RPQ was examined and patients were asked to fill missing data before leaving the outpatient clinic. There was no consistency in the items with omitted response. In all the cases, patients referred that they skipped it by mistake. No item was considered objectionable; however, two of the five male participants, agreed that the pregnancy related issues item was not applicable.

#### Item scoring and feasibility

Table 2 summarizes item scoring and t-scores as a result of the linear z transformation in the 270 patients which data were used for the validation process. In addition, (mean±SD) RPQ score for the sample was 50±6.69.

**Table 2:** Item description and scoring.

Dimension/Component	Item	Mean	SD	z*	t*
**I Likelihood to develop articular and extra-articular manifestations**					
1. Joint pain	I will always have pain	46.9	31.9	-0.06	49.4
2. Joint swelling	People who have rheumatoid arthritis will always have swollen joints	44.2	31.3	-0.04	49.6
3. Morning stiffness	How likely is it that I feel stiff and numb because I have rheumatoid arthritis?	53.1	30.7	0.09	50.9
4. Substantial fatigue	People who are suffering rheumatoid arthritis will always be exhausted	45.5	31.2	-0.10	494
5. Rheumatoid nodes	How likely is it that I will get lumps (nodules) in some part of my body because I have rheumatoid arthritis?	51.7	31.8	-0.05	49.5
6. Peripheral neuropathy	Surely at some point, I will feel cramps, tingling and burning in my feet, legs or arms	54.1	30.7	0.06	50.6
7. Vasculitis	I can expect to get spots because my veins are swelling and blood is not circulating properly	44.8	32.9	-0.08	49.2
8. Secondary Sjögren’s syndrome	I can expect my eyes and mouth to bother me because they feel dry	59.8	32.8	0.34	53.4
9. Atlantoaxial subluxation	People with rheumatoid arthritis will feel pain in the neck and the back of neck where the hump is	51.5	31.1	0.05	50.5
**II Likelihood to develop complications and/or comorbidities and disease severity**					
10. Ophthalmic	At some point I’m going to experience eye disease	58.1	29.4	-0.27	52.7
11. Cardiovascular	Heart disease is something that will probably happen to me	45.8	28.7	0.06	50.6
12. Pulmonary	I’m going to have lung disease at some point	42.6	29.8	-0.12	48.8
13. Cutaneous	Surely I will have skin problems at some point	50.6	31.7	-0.02	49.8
14. Reduction of life expectancy	Rheumatoid arthritis will kill me	37.3	32.4	-0.24	47.6
15. Polypharmacy	To treat my arthritis I will need to take many medications for a long time and this will probably cause me problems	68.6	27.3	0.24	52.4
16. Disease severity	How serious do you consider a disease such as rheumatoid arthritis?	62.6	27.2	0.22	52.1
**III Likelihood to develop socioeconomic unfavorable consequences**					
17. Disability or functional limitation	What is the probability that rheumatoid arthritis causes me disability?	62.4	28.9	0.31	53.1
18. Physical deformity	How possible is it that my fingers will become deformed because I have rheumatoid arthritis?	64.9	27.9	0.24	52.4
19. Joint replacement indication	How likely is it that my joints hurt and I will need to use an artificial joint? (prosthesis)	53.7	31.3	0.01	50.1
20. Depression	Feeling sad, discouraged and without a future is something that will happen to those of us with rheumatoid arthritis	44.1	32.6	-0.19	48.1
21. Dependence / loss of autonomy	Rheumatoid arthritis is a disease that will make me to depend on others and lose my autonomy	54.3	31.1	0.20	51.9
22. Loss of job or loss of financial security	How possible is it that I will lose my job or have an economic crisis because I am sick with rheumatoid arthritis?	56.9	32.4	0.40	54
23. Impact on social relations	How much will rheumatoid arthritis affect the relationships with our partners, family and / or friends in some way?	37.4	32.8	-0.38	46.2
24. Pregnancy related issues	Of every 10 women who get rheumatoid arthritis, how many will have problems getting pregnant or during pregnancy and will give birth to sick children?	43.4	33.2	-0.22	47.8
**IV Perception of personal responsibility to prevent and develop RA-related complications**					
25. Responsibility	How responsible am I for the complications I may have from rheumatoid arthritis?	19.7	26.6	-0.48	45.2
26. Prevention	I think that the complications I may have due to my illness can be prevented	25.1	29.9	-0.57	44.3
**V Perception of personal control over the disease**					
27. Disease control	How capable am I of controlling the discomfort and complications of my illness?	61.6	30.6	0.21	52.1

*Median of z & t scores

Finally, the RPQ was feasible, the mean of time required to fill it was of 13 minutes and all patients agreed the time was convenient. Eighty nine percent of the patients agreed about instructions and item´s semantic clarity and 97% of them agreed about adequacy of the RPQ format.

### Psychometric validation (Step 3)

#### Internal consistency and reliability

Results of internal consistency (Cronbach´s α) and reliability/test-retest (ICC and 95% CI) for each dimension of the RPQ are presented in Table 3, which additionally includes inter-item correlation and floor and ceiling effects.

**Table 3:** Psychometric characteristics of the RPQ by dimension.

RPQ Dimension	Cronbach´s α	ICC 95% CI	Mean of inter-item correlations	Floor effect/ceiling effect (%)
**Likelihood to develop articular and extra-articular manifestations (9 items)**	0.93	0.93 0.91–0.94	0.59	0 / 0.7
**Likelihood to develop complications and/or comorbidities and disease severity (7 items)**	0.86	0.86 0.81–0.89	0.52	0 / 0.7
**Likelihood to develop socioeconomic unfavorable consequences (8 items)**	0.89	0.87 0.84–0.90	0.51	0 / 0
**Perception of personal responsibility to prevent and develop RA-related complications (2 items)**	0.81	0.80 0.74–0.85	0.69	15.9 / 0
**Perception of personal control over the disease (1 item)**	NA	NA	NA	3.3 / 3.7

NA = not applicable; ICC = intraclass correlation coefficient; CI = confidence interval

The mean (±SD) of the time between the 2 measurements in the test-retest analysis was of 12.9 (±3.7) days.

Finally, the whole RPQ internal consistency was excellent (Cronbach´s α = 0.90) as was reliability/test-retest (ICC = 0.93; 95%CI: 0.90–0.95); floor and ceiling effects were of 0% each.

#### Validity

The final version of the RPQ was submitted for face and content validity; each of the 27 items had ≥80% agreement from experts, regarding the presence of the following item´s characteristics: Relevance (90%), adequate wording (81%) and appropriate language and meaning (85%). No modifications were proposed.

Construct validity was evaluated with factor analysis and results are summarized in Table 4. The KMO measure of 0.911 and significant result (*X*^2^ = 5540.24 p<0.001) for the Bartlett sphericity test confirmed the adequacy of the sample. A 5-factor structure was extracted, which accounted for 68.8% of the total variance. All factors had eigenvalues >1. Factors were equivalent to the 5 dimensions above described (Likelihood to develop RA-related articular and extra-articular manifestations, likelihood to develop RA-related complications and/or comorbidities and disease severity, likelihood to develop RA-related socioeconomic unfavorable consequences, patient perception of personal responsibility to prevent and develop complications and patient perception of personal control over the disease) (Tables 2–4 and Fig 2).

**Table 4:** Factor loadings for the five factors after Varimax rotation of the RPQ.

Dimension/Item	Factorial loadings				
	I	II	III	IV	V
**I Likelihood to develop articular and extra-articular manifestations**					
I will always have pain	.73				
People who have rheumatoid arthritis will always have swollen joints	.82				
How likely is it that I feel stiff and numb because I have rheumatoid arthritis?	.78				
People who are suffering from rheumatoid arthritis will always be exhausted	.66				
How likely is it that I will get lumps (nodules) in some part of my body because I have rheumatoid arthritis?	.69				
Surely at some point, I will feel cramps, tingling and burning in my feet, legs or arms	.62				
I can expect to get spots because my veins are swelling and blood is not circulating properly	.45				
I can expect my eyes and mouth to bother me because they feel dry	.49				
People with rheumatoid arthritis will feel pain in the neck and the back of neck where the hump is	.59				
**II Likelihood to develop complications and/or comorbidities and disease severity**					
At some point I’m going to experience eye disease		.67			
Heart disease is something that will probably happen to me		.81			
I’m going to have lung disease at some point		.79			
Surely I will have skin problems at some point		.60			
Rheumatoid arthritis will kill me		.53			
To treat my arthritis I will need to take many medications for a long time and this will probably cause me problems		.50			
How serious do you consider a disease such as rheumatoid arthritis?		.45			
**III Likelihood to develop socioeconomic unfavorable consequences**					
What is the probability that rheumatoid arthritis causes me disability?			.72		
How possible is it that my fingers will become deformed because I have rheumatoid arthritis?			.64		
How likely is it that my joints hurt and I will need to use an artificial joint? (prosthesis)			.53		
Feeling sad, discouraged and without a future is something that will happen to those of us with rheumatoid arthritis			.49		
Rheumatoid arthritis is a disease that will make me depend on others and lose my autonomy			.61		
How possible is it that I will lose my job or have an economic crisis because I am sick with rheumatoid arthritis?			.72		
How much will rheumatoid arthritis affect the relationships with our partners, family and / or friends in some way?			.52		
Of every 10 women who get rheumatoid arthritis, how many will have problems getting pregnant or during pregnancy and will give birth to sick children?			.58		
**IV Perception of personal responsibility to prevent and develop RA-related complications**					
How responsible I am for the complications I may have from rheumatoid arthritis?				.85	
I think that the complications I may have due to my illness can be prevented				.83	
**V Perception of personal control over the disease**					
How capable am I of controlling the discomfort and complications of my illness?					.83

Extraction method: Principal component analysis.

Rotation method: Varimax normalization with Kaiser.

Table 5 describes external criteria per dimension selected because they were recorded on the charts. No external criteria for dimension 5 (”Patient perception of his/her personal control over the disease”) was found convenient and consistently documented on the charts. The individual external criteria were more frequently present in the patients with RP than in their counterparts (but the external criteria: patient missed medical appointments, from the dimension 4: “Patient perception of personal responsibility to prevent and develop complications”); differences were significant for the majority of the external criteria selected (Table 5).

**Table 5:** External criteria per dimension and comparison of their presence between patients with and without RP.

Dimension/External criteria recorded from charts review	N° (%) of patients with RPQ score ≥ 61.7 mm	N° (%) of patients with RPQ score < 61.7 mm	p value
**Likelihood to develop articular and extra-articular manifestations (Dimension 1)**			
Joint pain^1^	31 (46.3)	18 (8.9)	0.000
Joint swelling^1^	90 (44.8)	9 (4.4)	0.000
Significant morning stiffness^1^	25 (37.3)	6 (3)	0.000
RA-related treatment intensification^1^	30 (44.8)	25 (12.3)	0.000
**Likelihood to develop complications and/or comorbidities and disease severity (Dimension 2)**			
Joint replacement indication^2^	35 (52.2)	37 (18.2)	0.000
Three or more (non-rheumatic) additional consultations^1^	39 (58.2)	101 (49.8)	0.260
Prior hospitalization^2^	14 (20.9)	30 (14.8)	0.255
Prior neck or hand surgery indication^2^	7 (10.4)	8 (3.9)	0.062
**Likelihood to develop socioeconomic unfavorable consequences (Dimension 3)**			
Intensive RA treatment^3^	25 (37.3)	49 (24.1)	0.041
**Patient perception of personal responsibility to prevent and develop RA-related complications (Dimension 4)**			
Missed medical appointments^1^	5 (7.5)	16 (7.9)	1
**(Mean±SD) N° of external criteria**	3.6 (2.4)	1.5 (1.3)	0.000

^1^ = if recorded during the year previous to RPQ application;

^2^ = if ever recorded on the charts;

^3^ ≥3 combined DMARDs and low-doses of corticosteroids.

Finally, logistic regression models consistently showed that patients with ≥3 external criteria (reference group) had an increased risk of RPQ score ≥61.7 mm; results were reproduced when patients from the reference group were compared to patients with 0, 1 and 2 external criteria as summarized in Table 6.

**Table 6:** Logistic regression analysis of RP (presence) based on number of external criteria.

	2 vs ≥ 3 external criteria	1 vs ≥ 3 external criteria	None vs ≥ 3 external criteria
**OR to RP**	2.307	2.934	8.730
**95% CI**	1.622–3.281	2–4.293	3.715–20.514
**R**^**2**^	0.296	0.269	0.278
**p value**	0.000	0.000	0.000

OR = odds ratio; RP = risk perception; CI = confidence interval

## Discussion

In the present study, we developed and validated the RPQ in Spanish speaking RA patients; standardized test-construction methods were used for its development and the final version showed adequate psychometric properties in terms of construct, content, criteria and face validity, and reliability, which was evaluated with internal consistency and test-retest, as recommended [31, 36–39]. The RPQ was also feasible based on patient´s evaluation and suitable for low-literacy patients; accordingly, its use may be more easily generalized. Patients were additionally involved in the RP-model construction and performed RPQ face validity; it should be emphasized that patient´s involvement is particularly relevant as one would expect the dimensions of RP to be defined by the patients themselves in addition to by health care professionals. Three different samples of consecutive RA outpatients, which were representative of real-world RA outpatients attending a tertiary care level center, were used to perform analysis.

The RPQ showed adequate internal consistency; Cronbach´s α coefficient for the total scale was excellent, with α value of 0.90 [35]. The test-retest reliability assessed in 50 patients by the same researcher showed an ICC of 0.93, indicating excellent reliability, with 95% CI of 0.90–0.95 [36]. The construct validity was demonstrated by KMO sampling and Barlett´s test of sphericity, both confirming the adequacy of the sample size for conducting factor analysis [37], and a single factor structure was extracted, accounting for 68.8% of the variance. Face and content validity were examined by a multidisciplinary group of experts involved in RA management, and patients confirmed RPQ feasibility. The positive association between the number of external criteria and a RPQ score above 61.7 mm (considered the cut-off to define presence of RP) favored criterion validity. The RPQ did not show neither floor nor ceiling effect. A five-factor model was most appropriate for the RPQ and the following factors (conceptually equivalent to the 5 dimensions derived from model construction) were used: 1) Likelihood to develop RA-related articular and extra-articular manifestations 2), Likelihood to develop RA-related complications, comorbidities and severity, 3) Likelihood to develop RA-related socioeconomic unfavorable consequences, 4) Personal responsibility to develop and prevent RA complications and 5) Patient perception of personal control over the disease. The five factors together explained almost seventy percent of the total variance.

The first 3 dimensions related to probability included joint and extra-articular manifestations, complications, comorbidities and unfavorable socioeconomic events, and they were rated as the most important by the patients and the rheumatology professionals. Patients are frequently unaware of medical and technical names, nonetheless, they can identified disease/treatment-related unpleasant experiences [42] and eventually embrace a patient-health care provider relationship where treatment plans are negotiated and shared. Finally, the 2 dimensions left were related to patient´s perception of his/her personal responsibility to develop, prevent and control disease-related-complications; these dimensions give a specific clinical context that facilitates patient´s adoption of treatment plans with the expectation to delay/avoid disease-related-damage; both are particularly relevant for the patient´s engagement to smoke cessation, weight control and exercising, and compliance with treatment, which are recognized as fundamental to achieve disease control and better outcomes.

The structure of the RPQ underwent an important modification from the initial conceptual model. We expected the severity dimension to be expressed independently, but after factor analysis, it was grouped to the likelihood of RA-related complications and comorbidities dimension. Patient and physicians have different perspectives about RA outcomes and this may be extended to the perception of the disease severity [40]. Traditionally, RA severity has been associated to the presence of erosions, structural damage and ultimately, radiographic progression and joint destruction; erosions are very characteristics of RA and, unless the disease is early and intensively treated, develop in the majority of the patients with long-standing disease. Erosions and radiographic progression are relevant because they translate into outcomes that matter to patients as increase work disability, increase necessity of surgical joint replacement and increase mortality [43–45]. All of them, may be perceived by the patients as disease complications. It is worth mentioning that theories dedicated to disease´s perception, describe that patient´s representations of a particular disease are integrated with their pre-existing schemes, which give sense to their symptoms. The theory also describes a parallel response model, in which patients process emotional responses to illness and cognitive representations of illness relatively independently. Both, cognition and emotional responses, provide motivation for people to adopt specific behaviors that ultimately improve (in the best scenario) disease´s outcomes [46]. Weinman et al [47] developed the Illness Perception Questionnaire (IPQ) which aimed to provide a quantitative assessment of the 5 conceptual components of illness representation; in their questionnaire, authors assigned disease severity to the consequences dimension (their questionnaire did not include a probability dimension) and factor analysis confirmed such assignment. Importantly, the IPQ has been studied in a wide variety of diseases, among them in 27 diseases of the musculoskeletal system and connective tissue diseases [48]. Only 2 studies had included RA patients. Moss-Morris and Chalder [49] compared illness perceptions and self-reported disability in 49 patients with chronic fatigue syndrome and 74 RA outpatients with long-standing disease; authors showed that patients with chronic fatigue syndrome had more negative views about their symptoms and a greater impact of them on their lives, when compared to RA patients. Hyphantis et al [50] compared psychological distress symptoms and illness perception in 55 ankylosing spondylitis Greek patients and 199 RA longstanding outpatients that served as controls; the authors found that in former patients, cognitive variables correlated with health related quality of life; meanwhile, in RA patients, depressive symptoms (present in 25.1% of the patients) and illness perception equally contribute to the health related quality of life. It needs to be emphasize that illness and risk perception differ in terms of the concept´s extension; meanwhile “illness perception” refers to the mental representations and personal ideas that people have about an illness, “risk perception” is limited to the negative consequences of a particular illness and need to be translated (but not limited to) in terms of probability [29, 48].

Some limitations of the present study included the lack of representation of the whole clinical *spectrum* of RA, which has been recognized as a complex syndrome with multiple clinical presentations. Also, patients recruited were representative of typical RA patients, from a tertiary care level center; they were primarily middle-aged females, with disease specific autoantibodies, long-standing disease, substantial comorbidity and disease under control; some of these characteristics may represent a more severe disease and patients may have scored higher in the RPQ; nonetheless, median RPQ score was around median values (61.7 mm) and patients with atypical characteristics such as males, patients who lack autoantibodies in their serum, patients with early disease and with higher levels of disease activity were also represented. The RPQ was constructed and validated in a particular population of Spanish speaking RA outpatients from Mexico with particular demographic and disease characteristics [40, 41]. Nonetheless, we consider its use could be extended to other Spanish speaking RA populations around the world. Finally, the RPQ responsibility and control dimensions were unintentionally located at the end of the questionnaire and it may have affected their scoring and bias results.

Current recommendations for RA patients emphasize the use of disease modifying anti-rheumatic drugs integrated in a self-care model, with regular visits to the rheumatologist, laboratory testing and sometimes, additional diagnostic procedures [9, 51]. Moreover, patients are frequently requested to adopt changes in their health-related behavior in order to achieve a healthy lifestyle and improve outcomes. Such complex intervention may be more successfully maintained when the primary physician embraces patient-centred-care. The Institute of Medicine defines patient-centred-care as: “Providing care that is respectful of, and responsive to, individual patient´s preferences, needs, and values, ensuring that patient´s values guide all clinical decisions” [52]. In such conceptual model of patient´s care (but not limited to), the RPQ can provide information to rheumatologists about the risks perceived by their patients, and may help them understand how ideas of the disease and the treatment (positively or negatively) influence patient self-care related behavior.

## Conclusions

The RPQ was constructed integrating both, patients and health care providers´ perspectives and was found to be valid, reliable and feasible to evaluate RP in our population. There is evidence in non-rheumatic diseases suggesting that RP is associated to better health related behavior, which may be conceived as a valuable outcome. If these results are confirmed in RA populations, the RPQ could be incorporated to routine clinical care and conceived as a positive (cognitive) intervention.

## Supporting information

S1 Appendix “Guide for semi-structured interviews”. (PDF)

S2 Appendix “Final draft of the RPQ”. (PDF)
