# Supplementary material for: Validation of a risk perception questionnaire developed for patients with rheumatoid arthritis
Source: PLoS One. 2019 Jul 22;14(7):e0219921. doi: 10.1371/journal.pone.0219921 (PMC6645517; doi:10.1371/journal.pone.0219921)
Supplement: S1 Appendix — (PDF) [file pone.0219921.s001.pdf]

Guide for semi-structured interviews during construction of risk perception of conceptual model step.

Spanish	English
Estimado paciente, ahora que está usted terminando su participación en el estudio de investigación clínica, quisiéramos platicar con usted acerca de las preocupaciones que pudiera tener debido a esta situación.	Dear patient, now that you are ending your participation in the clinical research study, we would like to discuss with you any concerns you may have about this situation.
¿Cómo se siente por estar terminando el estudio de investigación?	How does it feel to be finishing the research study?
¿Le preocupa dejar de tomar el medicamento que se le estuvo proporcionando durante el estudio de investigación?	Are you worried about stopping taking the medication that was being giving to you during the research study?
¿Si es así, cuáles son las cosas que le preocupan o le inquietan?	If so, what are the issues that concern or disturb you?
¿Por qué esas cosas son motivo de preocupación para usted?	Why are those issues, source of concern for you?
¿Cuál de todas ellas es la que más le inquieta y por qué?	Which of them is your main concern and why?
¿Si piensa en su futuro hay algo que le de miedo?	If you think about your future, is there anything that fears you?
**Nota para el entrevistador: En caso de que no haya sido mencionado por el paciente en la conversación previa, invitar al paciente a reflexionar acerca de los siguientes temas en el contexto clínico de un paciente con artritis reumatoide:*	** Note to the interviewer: In case it has not been mentioned by the patient in the previous conversation, invite the patient to think about the following items in the clinical context of the RA patient:*
Curso de la enfermedad	*Course of the disease*
Recaídas	*Flares*
Tratamiento	*Treatment*
Vida personal, familiar y social	*Personal, family and social life*
Trabajo	*Work*
Economía	*Economy*
