# Supplementary material for: Validation of a risk perception questionnaire developed for patients with rheumatoid arthritis
Source: PLoS One. 2019 Jul 22;14(7):e0219921. doi: 10.1371/journal.pone.0219921 (PMC6645517; doi:10.1371/journal.pone.0219921)
Supplement: S2 Appendix — (PDF) [file pone.0219921.s002.pdf]

CUESTIONARIO DE PERCEPCIÓN DE RIESGO EN ARTRITIS REUMATOIDE

Fecha__________________

Estimado paciente, este cuestionario tiene la intención de conocer su opinión con respecto a algunas situaciones relacionadas con la artritis reumatoide, por lo que la información que contiene no es necesariamente la verdad sobre esta enfermedad.

Le pedimos que a cada pregunta o afirmación, nos ayude a **marcar sobre la línea de abajo** el punto que mejor refleje su opinión.

Ejemplo: 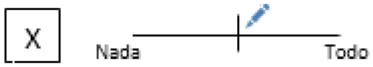

1 Seguramente siempre tendré dolor

Nada probable — **Totalmente** probable

2 Las personas que tenemos artritis reumatoide siempre tendremos las articulaciones inflamadas.

Nada probable — **Totalmente** probable

3 ¿Qué tan posible es que me sienta rígido y entumido por estar enfermo de artritis reumatoide?

Nada posible — **Totalmente** posible

4 Las personas que estamos enfermos de artritis reumatoide siempre estaremos agotados

Nada probable — **Totalmente** probable

5 ¿Qué tan posible es que me salgan bolitas (nódulos) en alguna parte del cuerpo por estar enfermo de artritis reumatoide?

Nada probable — **Totalmente** probable

6 Seguramente en algún momento sentiré calambres, hormigueos y ardor en mis pies, piernas o brazos

Nada probable — **Totalmente** probable

7 Puedo esperar que me salgan manchas porque mis venas se inflamaron y la sangre no circula adecuadamente

Nada probable — **Totalmente** probable

8 A las personas con artritis reumatoide, se nos lastimará el cuello y nos dolerá la parte de atrás, por donde sale la joroba

Nada probable — **Totalmente** probable

9 Puedo esperar que los ojos y la boca me molesten por sentirlos secos

Nada probable — **Totalmente** probable

10 En algún momento voy a enfermarme de los ojos

Nada probable — **Totalmente** probable

11 Enfermarme del corazón es algo que muy probablemente me pase

Nada probable — **Totalmente** probable

12 Me voy a enfermar de los pulmones en algún momento

Nada probable — **Totalmente** probable

13 Seguramente voy a tener problemas de la piel en algún momento

Nada probable — **Totalmente** probable

14 La artritis reumatoide me llevará a la muerte

Nada probable — **Totalmente** probable

15 Para tratar mi artritis necesitaré tomar muchos medicamentos durante largo tiempo y esto probablemente me cause problemas

Nada probable — **Totalmente** probable

16 ¿Cuál es la probabilidad de que la artritis reumatoide me lleve a tener discapacidad?

Nada probable — **Totalmente** probable

17 ¿Qué tan posible es que mis dedos se deformen debido a que tengo artritis reumatoide?

Nada posible — **Totalmente** posible

18 ¿Qué tan probable es que mis articulaciones se lastimen y necesite que me pongan una articulación artificial? (prótesis)

Nada probable — **Totalmente** probable

19 Sentirse triste, desanimado y sin futuro es algo que nos pasará a quienes tenemos artritis reumatoide

Nada probable **Totalmente** probable

20 La artritis reumatoide es una enfermedad que me llevará a depender de los demás y a perder mi autonomía

Nada probable **Totalmente** probable

21 ¿Qué tan posible es que pierda mi trabajo o pase por una crisis económica debido a que estoy enfermo de artritis reumatoide?

Nada posible **Totalmente** posible

22 ¿Qué tanto nos pasará a quienes tenemos artritis reumatoide, que las relaciones con nuestras parejas, familiares y/o amigos se verán afectadas de alguna manera?

Nada probable **Totalmente** probable

23 De cada 10 mujeres que se enferman de artritis reumatoide, ¿cuántas tendrán problemas para embarazarse o durante el embarazo y darán a luz hijos enfermos?

Ninguna **Todas**

24 ¿Qué tan responsable soy de las molestias y complicaciones que pueda tener por la artritis reumatoide?

Nada **Totalmente**

25 Creo que las molestias y complicaciones de la enfermedad que tengo, se pueden prevenir

Nada probable **Totalmente** probable

26 ¿Qué tan capaz me siento de controlar las molestias y complicaciones propias de mi enfermedad?

Nada **Totalmente**

27 ¿Qué tan grave considera usted una enfermedad como la artritis reumatoide?

Nada grave **Totalmente** grave

MUCHAS GRACIAS POR HABER CONTESTADO ESTE CUESTIONARIO
